# Myokines: metabolic regulation in obesity and type 2 diabetes

**DOI:** 10.1093/lifemeta/loae006

**Published:** 2024-03-02

**Authors:** Zhi-Tian Chen, Zhi-Xuan Weng, Jiandie D Lin, Zhuo-Xian Meng

**Affiliations:** Department of Pathology and Pathophysiology and Department of Cardiology of the Second Affiliated Hospital, School of Medicine, Zhejiang University, Hangzhou, Zhejiang 310058, China; Key Laboratory of Disease Proteomics of Zhejiang Province, School of Medicine, Zhejiang University, Hangzhou, Zhejiang 310058, China; Zhejiang University-University of Edinburgh Institute (ZJE), School of Medicine, Zhejiang University, Haining, Zhejiang 314400, China; Department of Pathology and Pathophysiology and Department of Cardiology of the Second Affiliated Hospital, School of Medicine, Zhejiang University, Hangzhou, Zhejiang 310058, China; Key Laboratory of Disease Proteomics of Zhejiang Province, School of Medicine, Zhejiang University, Hangzhou, Zhejiang 310058, China; Life Sciences Institute and Department of Cell and Developmental Biology, University of Michigan, Ann Arbor, MI 48109, United States; Department of Pathology and Pathophysiology and Department of Cardiology of the Second Affiliated Hospital, School of Medicine, Zhejiang University, Hangzhou, Zhejiang 310058, China; Key Laboratory of Disease Proteomics of Zhejiang Province, School of Medicine, Zhejiang University, Hangzhou, Zhejiang 310058, China; Department of Geriatrics, Affiliated Hangzhou First People’s Hospital, Hangzhou, Zhejiang 310006, China

**Keywords:** myokines, skeletal muscle, obesity, type 2 diabetes, metabolism

## Abstract

Skeletal muscle plays a vital role in the regulation of systemic metabolism, partly through its secretion of endocrine factors which are collectively known as myokines. Altered myokine levels are associated with metabolic diseases, such as type 2 diabetes (T2D). The significance of interorgan crosstalk, particularly through myokines, has emerged as a fundamental aspect of nutrient and energy homeostasis. However, a comprehensive understanding of myokine biology in the setting of obesity and T2D remains a major challenge. In this review, we discuss the regulation and biological functions of key myokines that have been extensively studied during the past two decades, namely interleukin 6 (IL-6), irisin, myostatin (MSTN), growth differentiation factor 11 (GDF11), fibroblast growth factor 21 (FGF21), apelin, brain-derived neurotrophic factor (BDNF), meteorin-like (Metrnl), secreted protein acidic and rich in cysteine (SPARC), β-aminoisobutyric acid (BAIBA), Musclin, and Dickkopf 3 (Dkk3). Related to these, we detail the role of exercise in myokine expression and secretion together with their contributions to metabolic physiology and disease. Despite significant advancements in myokine research, many myokines remain challenging to measure accurately and investigate thoroughly. Hence, new research techniques and detection methods should be developed and rigorously tested. Therefore, developing a comprehensive perspective on myokine biology is crucial, as this will likely offer new insights into the pathophysiological mechanisms underlying obesity and T2D and may reveal novel targets for therapeutic interventions.

## Introduction

Metabolic homeostasis is regulated by endocrine hormones released by diverse cell types in the body. These mediate the crosstalk among different organs and tissues, including skeletal muscle [1], adipose tissue [2], liver [3], and the nervous system [4, 5]. Emerging evidence has revealed that such secreted proteins exert their effects via their autocrine, paracrine, and/or endocrine functions [6, 7]. Studies have identified numerous adipose tissue-derived secreted proteins (adipokines) that play important roles in the regulation of metabolic homeostasis in adipose tissue itself as well as in other metabolic organs [2, 8, 9]. For example, under sustained metabolic stress, adipose tissue changes adipokine release, affecting the metabolism of carbohydrates and lipids [2, 10]. Clinical studies have reported that an altered adipokine profile is associated with obesity, type 2 diabetes (T2D), and some other metabolic diseases [11]. The liver is a key participator in the regulation of glucose production and storage (as glycogen), as well as various aspects of lipid metabolism, contributing to the pathogenesis of metabolic diseases including T2D and nonalcoholic fatty liver disease (NAFLD) [12]. Secreted factors from the liver (hepatokines) are thereby involved in key regulation processes, mediating the communication between the liver and other organs in the body as well as regulating the hepatic and systemic metabolism [13, 14]. Neuron-secreted proteins also participate in the regulation of cholesterol metabolism [15] and energy homeostasis [16]. These secreted factors link different organs, making the metabolism more complex.

**Figure 1:**
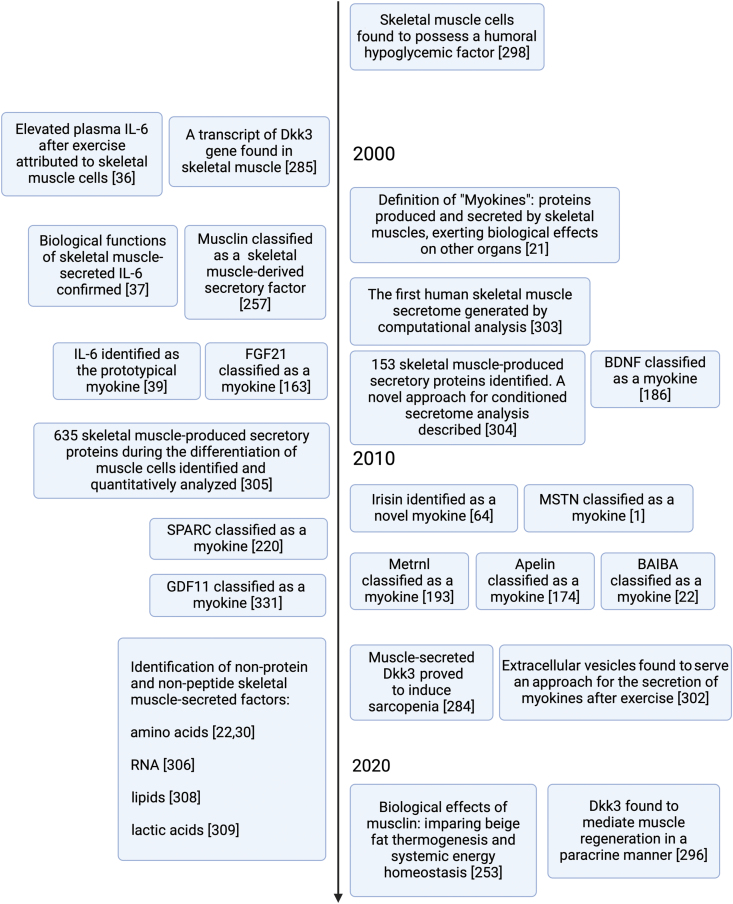
Milestones in myokine research. From the hypothesis of the humoral hypoglycemic factor in 1961, muscle-secreted factors, namely myokines, have been studied intensely over the past century. The classification of IL-6 as the first myokine was initially completed by the identification of its muscle source and corresponding biological effects. This led to the research on other novel myokines. IL-6, interleukin 6; MSTN, myostatin; GDF11, growth differentiation factor 11; Metrnl, meteorin-like; SPARC, secreted protein acidic and rich in cysteine; BAIBA, β-aminoisobutyric acid; FGF21, fibroblast growth factor 21; Dkk3, Dickkopf 3; BDNF, brain-derived neurotrophic factor. Created with BioRender.com.

As the largest organ in non-obese humans, skeletal muscle plays a pivotal role in whole-body nutrient and energy metabolism [1, 17–20]. Additionally, skeletal muscle is the main site of locomotion, being the primary site that mediates the impact of physical activity on body health [17]. In 2003, “myokines” were first defined as a kind of cytokine, being proteins and peptides secreted by the skeletal muscle that exert effects on other parts of the body [21]. Besides proteins and peptides, skeletal muscle also secretes small metabolites, such as amino acids [22]. As research has advanced, multiple myokines have been identified (Fig. 1). Numerous subsequent studies then revealed the abundant systemic effects of these myokines, demonstrating that the endocrine aspect of skeletal muscle deserves increased consideration [6]. Myokines released by skeletal muscle affect multiple metabolic and physiological pathways within skeletal muscle tissue *per se*, and in both adjacent and remote tissues and organs [23, 24]. In particular, myokines regulate the mass and browning of adipose tissue as well as gluconeogenesis and lipogenesis in the liver [1, 25]. Some myokines also participate in metabolic control of the brain, benefiting brain health [26, 27]. Myokines also act in concert with other crosstalk molecules, though this aspect requires further study [28].

Myokines secreted by skeletal muscle partly explain the beneficial effects of exercise on a wide variety of diseases, including cancer, cardiovascular diseases (CVDs), and diabetes [29, 30]. In response to nutrients and stress, skeletal muscle secretes myokines, which are then found to participate in physiological processes, including the metabolism of lipids and glucose, and the browning of white adipose tissue (WAT), indicating that they are important in the development of obesity and diabetes [6, 31]. The risk of T2D is positively correlated with excessive body fat mass, indicating the close relationship between obesity and T2D [32]. According to the International Diabetes Federation, about 483 million adults globally were living with T2D in 2021. T2D is identified by a progressive loss of insulin secretion and impaired insulin sensitivity, referred to as insulin resistance [33, 34]. A cure for T2D is still currently unavailable, which calls for a deeper understanding of the pathophysiology of the disease [35]. Studies of myokines are offering clues to how exercise benefits metabolism, promoting health conditions in patients with obesity and T2D.

In this review, we discuss several representative myokines in muscle-centered interorgan crosstalk, including interleukin 6 (IL-6), irisin, myostatin (MSTN), growth differentiation factor 11 (GDF11), fibroblast growth factor 21 (FGF21), apelin, brain-derived neurotrophic factor (BDNF), meteorin-like (Metrnl), secreted protein acidic and rich in cysteine (SPARC), and β-aminoisobutyric acid (BAIBA). We also summarize two myokines, Musclin and Dickkopf 3 (Dkk3), highlighting the discovery of their novel functions by our research group. By reviewing the role of these representative myokines, we aim to emphasize the endocrine function of skeletal muscle and its regulatory role on other metabolic organs in obesity and diabetes. These new findings provide new insights into the molecular mechanisms of obesity and T2D. Using our investigations on Musclin and Dkk3 as examples, we would like to highlight recent advances in myokine research owning to the application of new research technologies. More importantly, we discuss several challenges in current myokine research and potential solutions and future directions.

## IL-6

Whilst IL-6 is a well-known inflammatory factor closely related to the immune system, as a myokine, it also regulates diverse metabolic processes. Skeletal muscle has been identified as the primary source of elevated plasma IL-6 following exercise [36]. In 2004, skeletal muscle-derived IL-6 was demonstrated to regulate glucose homeostasis [37]. Compared to pathological conditions, it has been found that the increase in IL-6 levels in response to exercise intervention is not related to other immune-related proteins such as tumor necrosis factor α (TNF-α), suggesting that the function of IL-6 as a myokine may be distinct from its role in mediating inflammatory signaling [38, 39]. Intriguingly, by using the indocyanine green infusion technique to calculate net hepatosplanchnic IL-6 balance, it was found that the rise of IL-6 in blood circulation is restricted by the liver through its clearance effect, and this clearance mechanism is strengthened during exercise. This removal mechanism in the liver also seems to imply the importance of IL-6 in regulating metabolism [40].

There are three signaling pathways induced by IL-6. In classical signaling, IL-6 forms complexes by binding to IL-6 receptors (IL-6Rs) that exist in the cell membranes, leading to the activation of downstream transmembrane protein glycoprotein 130 (gp130) [41]. In trans-signaling, soluble IL-6 receptors (sIL-6Rs) bind to free IL-6 and further activate the downstream gp130 protein [42]. In cluster signaling, this mostly occurs in the cellular transmission of the immune system, the IL-6–IL-6R complex being transmitted by transmitting cells to cells that express gp130. For example, dendritic cells (DCs) can present IL-6–IL-6R complexes to T cells to inhibit the production of Th17 cells [43].

### Skeletal muscle

IL-6 exerts multiple regulatory functions on skeletal muscle and peripheral adipose tissue. Previous research has found that IL-6 can promote muscle hypertrophy [44, 45], lipolysis [46], and glucose uptake [47] in skeletal muscle. On the contrary, the inhibition of lipolysis can induce a compensatory increase of IL-6 in plasma [48]. In a recent study, it was found that Piezo1/Krüppel-like factor 15 (KLF15)/IL-6 axis induced muscle atrophy and antibodies against IL-6 could protect muscle from atrophy [49]. Acute IL-6 treatment was demonstrated to increase fatty acid oxidation and glucose uptake both *in vitro* and *in vivo*. This regulation of lipid and glucose metabolism can be induced by IL-6 through the adenosine monophosphate (AMP)-activated protein kinase (AMPK) pathway [50]. At the same time, glucose intake during exercise can reduce the increase in plasma IL-6 levels induced by exercise [51]. That is why IL-6 is also known as an energy sensor that is sensitive to the metabolic environment [38, 52]. Chronically elevated systemic IL-6 can disrupt mitochondrial functions and strength, and cause fatigue in skeletal muscle [53], which is also supported by a study on the aorta, showing that aging increases IL-6 levels and damages mitochondrial function [54].

### Other organs

IL-6 treatment on adipocytes triggers a decrease in ATP production, an increase in intracellular reactive oxygen species (ROS) levels, and changes in mitochondrial morphology [55]. However, some studies have pointed out that IL-6 is not necessary for maintaining mitochondrial content in adipose tissue *in vivo* [56], suggesting that there is still controversy over whether IL-6 regulates metabolism such as insulin sensitivity by regulating mitochondrial function.

IL-6 also regulates cardiac muscle. Given that lipids are an important source of energy, the high demand for energy in cardiac work is closely related to the regulation of lipid metabolism by IL-6. Under pathological conditions, cardiac lipotoxicity often leads to cardiac dysfunction [57]. Previous studies seem conflicting in demonstrating the roles of IL-6 in the heart. On one hand, research has shown that IL-6 alleviates oxidative stress induced by lipopolysaccharide (LPS) in myocardial cells, suggesting that IL-6 may have protective functions [58]. On the other hand, IL-6 may act as an important pathogenic mediator to promote myocardial hypertrophy and fibrosis [59]. In the field of heart transplantation, inhibiting IL-6 signaling can mitigate immune rejection responses [60]. These completely opposing regulatory functions of IL-6 are closely related to the timing of its upregulation in the heart. Specifically, in the short term, the pro-inflammatory response induced by IL-6 can protect host cells, but long-term elevation of IL-6 can lead to chronic inflammation [61].

The impact of IL-6 on metabolism is also systemic. Treatment with recombinant human IL-6 promotes cortisol secretion, which in turn inhibits neutrophil infiltration into tissues and induces anti-inflammatory effects [62]. At the same time, IL-6 seems to be associated with increased insulin secretion by upregulating the secretion of glucagon-like peptide-1 (GLP-1) hormone in the small intestine. This illustrates another IL-6-mediated signaling way to control metabolic homeostasis [63].

In summary, besides serving as an inflammatory factor, IL-6 is also a classic and widely studied myokine. Currently, numerous articles have reported that IL-6 has endocrine effects on various organs. IL-6 participates in the regulation of various metabolic organs, including skeletal muscle, liver, and adipose tissues, although the specific effects require further investigation. The upregulation of IL-6 levels is often accompanied by muscle hypertrophy and mitochondrial dysfunction. Acting as an energy receptor, IL-6 plays a crucial role in enhancing glucose uptake and lipolysis.

## Irisin

In 2012, Boström *et al*. first identified irisin in cultured C2C12 myotubes as a target of peroxisome proliferator-activated receptor γ (PPARγ) coactivator-1 α (PGC-1α), which is a key regulator of mitochondrial biogenesis and energy metabolism. They found that upregulated PGC-1α expression in mice promotes the expression of fibronectin type III domain-containing protein 5 (FNDC5), which is later cleaved and secreted as the 112 amino acid protein, irisin [64]. The cleavage mechanisms have been poorly studied, and a disintegrin and metalloproteinase (ADAM)-10 might be the enzyme responsible for irisin cleavage [65]. After arriving at the target cell, irisin acts through integrin αV. Utilizing mass spectrometry and cryo-electron microscopy (cryo-EM), one recent study has found that muscle secretes extracellular heat shock protein 90α (eHsp90α) upon exercise, which, in turn, activates integrin αVβ5, enabling irisin to bind and signal through Hsp90α/αV/β5 complexes [66]. Irisin has been known for its ability to induce the brown-fat-like development of WAT [64]. It also promotes mitochondrial biogenesis, regulates oxidative metabolism, and decreases ROS/reactive nitrogen species production and inflammatory responses [67, 68].

In mice, skeletal muscle produces about 72% of the total circulating irisin with adipose tissue contributing to the rest [69]. In humans, FNDC5 is predominantly expressed in skeletal muscle compared to other tissues, such as the liver, bone, and adipose tissue [70, 71]. Compared to skeletal muscle, human adipose tissues produce 100–200 times lower FNDC5 protein [72]. However, measurements of circulating irisin levels in human plasma vary wildly in different studies, likely due to the uncertain factors of reliability between different detection methods [73, 74]. Furthermore, humans use the start codon ATA for FNDC5, whereas mice use ATG. ATA start codon is generally associated with low mRNA translation efficiency, suggesting that FNDC5 and irisin might be low in humans [69]. A later review discussed this conflict thoroughly but failed to discover why ATA is active enough to produce detectable levels of circulating FNDC5 and irisin in humans [75].

Irisin levels are affected by various factors, while their effects remain unclear. Exercise is one of the main factors that is reported to increase irisin levels. The production of irisin is upregulated by skeletal muscle contraction, which increases the level of PGC-1α, promoting the cleavage of FNDC5 and hence increasing the production of irisin [67, 76]. In addition, exercise-induced deprivation of intracellular muscle ATP may also promote the expression of FNDC5 and the production of irisin. Conversely, small mother against decapentaplegic (SMAD) family member 3 (SMAD3) suppresses the production of irisin by binding to the promoter regions of *Fndc5* and *PGC-1α*, reducing their transcription [69]. Most related studies have demonstrated that exercise increases both FNDC5 expression and irisin levels [67]. However, exercise-mimicking experiments cannot trigger irisin release from muscle *in vitro*, further illustrating the complex regulatory nature of irisin production [77]. One meta-analysis also showed declined irisin levels after chronic exercise, which might indicate different effects of acute and chronic exercise [78]. Bao *et al*. proposed that the secretion of irisin is not proportional to the expression of FNDC5, a bout of acute exercise increases irisin secretion by upregulated cleavage of FNDC5, and FNDC5 expression is elevated by long-term exercise [79].

In addition to exercise, some miRNAs are potential regulators for irisin. For example, c-miRNA-140 expression probably triggers less weight loss in response to diet and exercise interventions by suppressing the activity of FNDC5 and hence reducing the release of irisin [80].

Additionally, many studies have reported that irisin levels decrease with age [81], and are negatively correlated with insulin sensitivity [82] and adiponectin levels [83], suggesting related metabolic roles. In T2D patients, circulating irisin levels are also decreased [84, 85], and exercise, correspondingly, increases irisin levels [86]. Overall, regulated by various mechanisms, irisin affects a wide variety of organs, influencing a range of metabolic activities.

### Adipose tissue

One of the biological functions of irisin is its ability to promote brown fat formation, leading to increased energy expenditure and improved metabolic parameters [64, 69]. Hence, it is a promising therapeutic target for many diseases and disorders including T2D [64]. Irisin also induces phosphorylation of the extracellular signal-related kinase (ERK) and p38 mitogen-activated protein kinase (p38 MAPK) signaling pathways. These mediate the upregulation of mitochondrial uncoupling protein 1 (UCP1) in WAT. Upregulated UCP1, a thermogenic regulator in brown adipose tissue (BAT), contributes to the induction of white-to-brown shift [64, 87]. Mechanistically, irisin stimulates focal adhesion kinase (FAK), which promotes the ubiquitination of E3 ubiquitin-protein ligase WW domain-containing protein 2 (Wwp2), activating runt-related transcriptional factors 1/2 (RUNX1/2) and thus activating thermogenesis-related genes. In this Wwp2-dependent process, the PR domain-containing protein 16 (Prdm16) forms a complex with RUNX1/2, which is essential for the brown-fat-like development of white adipocytes [88]. By binding to the Hsp90α/αV/β5 complex, irisin triggers FAK phosphorylation, which is essential for the proliferation of adipose progenitor cells, a process required for brown adipocyte proliferation [89].

Despite these previous results mainly observed in mice, the extent to which irisin contributes to white fat browning in humans remains a matter of controversy [75, 90]. In human cell models, irisin failed to induce browning-related genes in the major WAT depots [75, 91, 92]. Further *in vivo* human-based studies are therefore required to reveal the actual role of irisin on WAT browning in a human context [69].

In addition to promoting the white-to-brown shift, irisin also influences adipose tissues in other ways. The adipogenic differentiation of adipose progenitor cells is suppressed by irisin treatment, indicating its ability to interfere with adipogenesis, contributing to decreased fat mass [93, 94]. By global RNA sequencing, irisin was found to upregulate the nuclear factor-kappaB (NF-κB) pathway, which in turn promotes the expression of C-X-C motif chemokine ligand 1 (CXCL1) predominantly in differentiated adipocytes. *CXCL1* is one of the genes that are upregulated in response to stimulation of heat production [92].

### Skeletal muscle

Many studies have shown that irisin facilitates metabolic abilities in skeletal muscle. In insulin-resistant mouse myoblast cells, irisin activates the p38/MAPK-PGC-1α axis, which sustains glucose uptake ability and promotes mitochondrial activity, improving insulin sensitivity [95]. Correspondingly, promoting irisin expression improved the viability of mouse myoblast cells under high glucose stress, as well as preserving the abilities of glucose uptake, glycogen accumulation, and fatty acid β-oxidation in response to insulin stimulation. Phosphorylation of AMPKα/insulin receptor β-subunit/ERK1/2 also contributes to the regulation of these effects, which is maintained by irisin [96, 97]. In human muscle cells, irisin promotes glucose and lipid metabolism by AMPK phosphorylation in an exercise-dependent manner [86]. However, little further evidence of the effects of irisin on human skeletal muscle has emerged in recent years.

### Pancreatic islets

Irisin activates protein kinase B (AKT)/B cell lymphoma 2 (BCL-2) signaling, contributing to the protection of both human and rodent β-cells and islets from palmitate-induced apoptosis. In mice and rats, irisin also improves insulin secretion [98]. However, there remain no evident associations between irisin levels and β-cell functions in T2D patients [99], with one study finding that irisin does not affect insulin secretion in human pancreatic islets [100]. As such, this signaling pathway needs to be further investigated and clarified in human cells.

### The liver

In a diabetic mouse model, irisin activates the phosphatidylinositol 4,5-bisphosphate 3-kinase (PI3K)/AKT/forkhead box transcription factor O1 (FoxO1) signaling pathway, which reduces the expression of phosphoenolpyruvate carboxykinase (PEPCK) and glucose-6-phosphatase (G6Pase), resulting in decreased gluconeogenesis in the liver. Irisin also increases glycogenesis by inducing PI3K/AKT/glycogen synthase kinase 3 (GSK-3)-mediated glycogen synthase [97]. Additionally, irisin improves lipid metabolism. In hepatocytes from obese mice, irisin inhibits cholesterol synthesis by AMPK-sterol regulatory element-binding transcription factor 2 (SREBP2) signaling [101]. Through binding with myeloid differentiation factor 2 (MD2) instead of toll-like receptor 4 (TLR4), irisin disturbs the formation of the MD2-TLR4 complex, interfering with MD2 recognition of stimuli including palmitic acid in diabetic individuals [102]. In addition, irisin helps maintain mitochondrial homeostasis by inhibiting the expression of mitochondrial fission-related proteins and promoting the expression of mitochondrial biogenesis-related proteins. Irisin also increases the expression of uncoupling protein 2 (UCP2), alleviating oxidative stress in human liver cells [103]. *Ex vivo* studies have found that irisin ameliorates dysregulated glucose and lipid metabolism, and improves hepatic insulin resistance and cell survival under high glucose-high insulin conditions [104].

To conclude, upregulated by physical activities, irisin promotes the white-to-brown shift of adipose tissue, increasing energy expenditure. Irisin is also known to enhance metabolism in skeletal muscles and provide protection to β-cells and the liver. Precise quantification of irisin is still an unsolved question and important for function analysis. In addition to gathering evidence from model organisms, many proposed functions need further confirmational studies in human cells.

## MSTN

Myostatin, also known as GDF8, has been identified in a molecular screen for new transforming growth factor β (TGF-β) superfamily members. Studies found that MSTN is expressed in skeletal muscle, which negatively regulates muscle mass [105]. In addition to the highly conserved sequence of MSTN in different animals, the role that MSTN functions as a negative regulator in muscle mass is also highly conserved [106, 107]. MSTN signals through activin type IIB receptors (ActRIIB), suppressing the growth and differentiation of myoblasts and preadipocytes [108, 109]. MSTN also inhibits the progression of cell cycles in resident muscle myoblasts [110, 111]. In contrast, inactivation of the *Mstn* gene doubles muscle mass in mice by promoting muscle fiber hypertrophy and hyperplasia [105]. At the organelle level, MSTN results in metabolic alterations of mitochondria and can induce mitochondria-dependent apoptosis in cancer cells. Under MSTN treatment, hexokinase II (HKII) is downregulated and dissociated from mitochondria, and voltage-dependent anion channel 1 is upregulated, increasing the translocation of Bax from the cytosol to mitochondria [112].

Like other TGFs, *Mstn* is first translated into a precursor protein. After maturation, MSTN is released and can act in either a paracrine or an autocrine manner. In obese humans and mice, MSTN secretion and plasma levels are increased, which may contribute to the systemic deterioration of skeletal muscle energy metabolism, leading to the progression to T2D [113, 114]. In skeletal muscle, the mRNA expression of *Mstn* is decreased after acute as well as long-term exercise, and its expression level is negatively correlated with insulin sensitivity [115]. Pharmacological inhibition of MSTN was found to improve systemic insulin sensitivity and whole-body glucose metabolism, indicating a potential therapeutic target for metabolic diseases [116].

### Adipose tissue

MSTN blocks the adipogenetic differentiation of brown adipocytes. Therefore, inhibition of MSTN and its receptor ActRIIB increases the amount of BAT and hence elevates energy expenditure [117, 118]. In addition, in WAT with MSTN deficiency, a BAT-like phenotype and gene expression profile could be observed, which may be due to enhanced cyclooxygenase-2 (COX-2) expression in these adipose cells [119]. This process could partly contribute to increased basal metabolic rate and O_2_ consumption rate in mice treated with an MSTN inhibitor [120, 121]. However, another study found that *Mstn* loss in mice results in lower rates of total and resting O_2_ consumption compared with the control group [122]. These studies considered body weight, namely the data were expressed as functions of body weight due to the body weights of mice with MSTN deficiency being higher than wild-type mice, both in males and females [123]. In addition, *Mstn* deficiency reduces fat accumulation and partially suppresses abnormal glucose metabolism. Compared to *Mstn*^+/+^ mice, *Mstn*^–/–^ mice exhibit markedly reduced total body fat mass and adipose cell size in the gonadal fat pad. The serum triglyceride, serum cholesterol levels, and triacylglycerol accumulation in WAT are also reduced upon inactivation of *Mstn* [119, 122].

Lack of MSTN induces enzymes participating in lipolysis and fatty acid β-oxidation in mitochondria of peripheral tissues, reducing fat accumulation [119]. Adipocytes from WAT in *Mstn*^−/−^ mice are consistently smaller than that in WT mice, whereas adipocyte hypertrophy caused by high-fat diet (HFD) feeding is not significantly different from that of WT mice. This could be attributed to reduced triacylglycerol synthesis or enhanced fatty acid oxidation [119]. In Meishan pigs, researchers found that MSTN regulates fatty acid metabolism via the myogenic transcription factor 2C (MEF2C)/miR222/stearoyl-CoA desaturase 5 (SCD5) pathway, thereby affecting fat deposition [124]. One study found that MSTN inhibits an adipogenesis-related transcription factor and genes participating in lipid metabolism, demonstrating its importance in fat accumulation [125].

### Skeletal muscle

In primary human muscle cells, MSTN promotes insulin-independent glucose uptake and enhances glucose oxidation and lactate production [115]. One study showed that MSTN promotes glucose uptake and consumption, increases glycolysis, and inhibits glycogen synthesis. Mechanistically, MSTN activates AMPK to promote glycolysis [126]. However, some other studies have demonstrated that MSTN inhibition in mice produces systemic metabolic benefits by increasing the anabolic effects in muscle, which increase insulin sensitivity, glucose uptake, and glycogen storage [106, 127, 128]. In this way, it seems that there remain conflicts on the effects of MSTN on glucose uptake, and further studies using advanced techniques are required to solve such discrepancies.

MSTN also participates in mitochondrial activities. Loss of *Mstn* in mice leads to interfered mitochondrial functions, including the tricarboxylic acid cycle (TCA) cycle, adenosine triphosphate (ATP) synthesis, oxidative phosphorylation, and thermogenesis [123, 129]. Mechanistically, it is achieved by downregulated AMPK/silent information regulator 1 (SIRT1)/PGC-1α signaling pathway in *Mstn*-knockout (KO) mice [123].

The metabolic regulatory roles of MSTN are mediated by other molecules. Further studies may reveal the molecular pathways and offer potential therapeutic targets. Some of these have already been elucidated. For example, MSTN inhibition leads to downregulated MSS51 mitochondrial translational activator, also named zinc finger MYND domain-containing protein 17 (Zmynd17), a mammalian skeletal muscle-specific protein localized to the mitochondria. The disruption of MSS51 expression promotes glycolysis, ATP levels, β-oxidation, and oxidative phosphorylation. *Mss51*-KO also increases oxygen consumption in myofibers, accelerating the skeletal muscle metabolism rate [130]. Hence, it is reasonable to speculate that the benefits of MSTN loss are partly mediated by MSS51. Furthermore, MSTN inhibits the expression of FNDC5 in a miR-34a-dependent manner. This partly explains enhanced thermogenic gene expression in *Mstn*^−/−^ adipocytes [131].

Additionally, MSTN is also regulated by other secretory proteins. In HFD-induced obese mice, overexpression of a hepatokine follistatin neutralizes MSTN and activates mechanistic target of rapamycin complex 1 (mTORC1) in skeletal muscle. This process promotes pathways of local nutrient uptake and energy expenditure, as well as a decline in adipose mass [132].

### The liver

MSTN signaling leads to phosphorylation of AMPK, which induces glucose uptake in the liver [133]. This process contributes to leucine-mediated hepatic glucose uptake. Suppression of miRNA-143 reduces the expression of MSTN [133].

In summary, MSTN is downregulated by exercise, promoting the differentiation of brown adipocytes. MSTN depletion also benefits glucose metabolism in adipose tissue, skeletal muscle, and liver. Some of the MSTN signaling pathways are related to miRNA, which could be further explored.

## GDF11

Growth differentiation factor 11, also known as bone morphogenetic protein 11 (BMP11), was first identified in rat incisor pulp RNA [134]. GDF11 belongs to the TGF‐β subfamily and exhibits variable levels of expression across different tissues [135]. Sharing a high similarity with other TGF-β family members such as MSTN, GDF11 is also secreted by skeletal muscle and regulates various metabolic pathways [136, 137].

In recent studies, GDF11 has been regarded as a potent “anti-aging” factor that promotes a calorie restriction‐like phenotype and improves glucose metabolism [138, 139]. Overexpression of the *Gdf11* gene prevents the manifestations of obesity and T2D in mice, including HFD-induced weight gain, hyperglycemia, insulin resistance, and glucose intolerance [139, 140]. Administration of exogenous GDF11 also has similar effects [141]. Sustained expression of the *Gdf11* gene induces AMPK activity, which is important in glucose uptake and homeostasis, and thus improves insulin resistance and glucose intolerance induced by HFD [140]. In addition, GDF11 participates in the regulation of many obesity-related signaling pathways, including SMAD, AKT, and p38 MAPK, indicating its importance in metabolism-related diseases, such as obesity, fatty liver, and T2D [139]. In both mice and humans, the expression levels of the *Gdf*11 gene are upregulated by exercise [137, 142].

Some studies have shown that the systemic GDF11 levels are decreased in T2D compared to normal conditions [143, 144]. Conversely, other researchers found that circulating GDF11 remains unchanged in T2D and obesity [145]. Moreover, some groups revealed higher plasma GDF11 levels in T2D patients, indicating a higher risk of the development of diabetes [146, 147]. Such contradictory results might be due to the similarity between the structures of MSTN and GDF11. However, this requires further validation.

### Adipose tissue

GDF11 triggers different signaling pathways in adipogenesis. In pre-adipocytes, GDF11 induces activin-like kinase 5 (ALK5)-SMAD2/3 activation, in cooperation with the Wingless/integration-1 (Wnt)/β-catenin pathway, resulting in suppressed differentiation from pre-adipocytes into adipocytes [139, 148]. One later study revealed that ALK5 is a functional receptor of GDF11 and GDF11 inhibits the expression of a transcription factor KLF15, which is an important adipogenic factor and an inhibitor for the Wnt/β-catenin pathway [149]. In mature adipocytes, GDF11 activates the Wnt pathway, regulating adiponectin secretion and insulin sensitivity [139].

In WAT, GDF11 treatment in obese mice results in a decrease in the size of adipocytes [139]. GDF11 overexpression also triggers PI3K/AKT/FoxO1, TGF-β/SMAD2, and AMPK signaling pathways by elevating phosphorylation levels of AKT, FoxO1, SMAD2, and AMPK [140]. In BAT, *Gdf11* gene transfer in obese and diabetic mice increases energy expenditure and oxidation by increasing the expression of *Ucp1* and *Ucp2*, which elevates the conversion of food energy to heat, preventing weight gain. In addition, *Elovl3*, another thermogenesis-related gene that plays an important role in BAT lipid recruitment, is also upregulated by GDF11. These mechanisms largely contribute to the prevention of HFD-induced weight gain and metabolic disorders [140].

### Skeletal muscle

Interestingly, exogenous GDF11 negatively regulates lipid metabolism in skeletal muscle, which is opposite to its effect in adipose tissue. In muscle cells, GDF11 treatment results in a decline in fatty acid transport protein levels, including the cluster of differentiation 36 (CD36) and fatty acid binding proteins, hence inhibiting fatty acid uptake of muscle cells. GDF11 also enhances the SMAD 2/3 pathway, downregulating vital enzymes that participate in intramyocellular triglyceride decomposition [150].

### Pancreatic islets

Studies have found that administration of exogenous GDF11 protein or overexpression of the *Gdf11* gene can promote function, morphology, and survival of β-cells and hence improve glucose metabolism in T2D mice [140, 143]. Systemic replenishment of GDF11 in diabetic mice increases the expression levels of v-maf musculoaponeurotic fibrosarcoma oncogene homolog A (*MafA*), pancreatic and duodenal homeobox factor-1 (*PDX-1*), and NK6 homeobox 1 (*NKX6.1*) in diabetic islets. These genes work together to synergistically promote insulin production and secretion, which are suppressed in T2D β-cells. This explains how GDF11 treatment restores glucose-stimulated insulin secretion (GSIS) [143].

In addition, GDF11 elevates the expression level of the antiapoptotic protein BCL-2 and reduces the expression of proapoptotic proteins Bax and cleaved-caspase3. In this way, GDF11 preserves β-cells by preventing hyperglycemia-induced apoptosis, but not by inducing proliferation [143]. Additionally, GDF11 also acts on α-cells in mouse models, reducing glucagon secretion [143].

### The liver

Sustained expression of the *Gdf11* gene changes the expression of genes that participate in lipid metabolism in HFD-fed animals, including the fatty acid translocase gene (*Cd36*), which may contribute to reduced serum concentrations of triacylglycerol, total cholesterol, and free fatty acid in HFD-fed mice. This process blocks fat accumulation and prevents fatty liver in HFD-fed mice. In obese mice, GDF11 ameliorates fatty liver by improving glucose intolerance and insulin resistance, leading to reduced hepatic steatosis. GDF11 also suppresses the expression of the gluconeogenesis gene *G6P* by acting on the PI3K/AKT/FoxO1 signaling pathway. This probably contributes to lower blood glucose levels and improved glucose homeostasis [140].

### The bone

It is likely that GDF11 negatively affects bone mass by regulating bone remodeling, one important aspect of osteoporosis. One related study similarly revealed a negative correlation between GDF11 and bone mineral density in postmenopausal Chinese women [151]. GDF11 has been revealed to activate SMAD2/3 and c-Fos-dependent induction of nuclear factor of activated T cells cytoplasmic 1 (Nfatc1), thereby stimulating receptor activator of NF-κB ligand (RANKL)-induced osteoclastogenesis, with GDF11 treatment also resulting in reduced expression of master osteogenic transcription factors *Runx2*, as well as alkaline phosphatase (*Alp*), Osterix (*Osx*), and *Osteocalcin* (*Ocn*), and osteoblast differentiation being inhibited. Bone loss also occurs in mice treated with GDF11 [152]. Interestingly, GDF11 and MSTN seem to have a similar role in bone metabolism [151].

GDF11 also participates in epigenetic processes. Elevated circulating GDF11 upregulates fat mass and obesity-associated protein (FTO), an RNA demethylase, in a CCAAT enhancer-binding protein alpha (C/EBPα)-dependent manner. FTO then targets PPARγ, shifting the fate of bone mesenchymal stem cells to adipocytes and thus inhibiting bone formation during osteoporosis [153].

Despite these findings, one recent study suggested an opposing view where upregulated GDF11 was suggested to activate the BMP signaling pathway, which enhances osteogenesis. These latter researchers concluded that in contrast to GDF11, MSTN negatively regulates bone mass [154]. GDF11 can probably function through all these pathways, but its final effect on bone metabolism requires further investigation.

In brief, physical activities induce increased GDF11 levels, inhibiting the differentiation of adipocytes and promoting thermogenesis. Additionally, GDF11 protects β-cells and the liver, improving glucose tolerance. The effects of GDF11 in bone suggest its potential role in epigenetic regulation, probably also in other tissues, which can be explored by further studies.

## FGF21

Fibroblast growth factor 21 is the 21st discovered *Fgf* gene in the FGF family [155]. FGF21 performs its function by activating FGF receptor 1c (FGFR1c) with cofactor β-Klotho (KLB) [156]. FGF21 is expressed in many secretory organs, especially in the liver, adipose tissue, and muscle [157]. In one classic past research example, FGF21 was found to activate PPARα to upregulate glucose uptake as a hepatokine [158]. Numerous studies have found that cold exposure can induce adipose tissues to secrete FGF21 and then enhance the browning of WAT to promote heat production [159]. FGF21 also stimulates fatty acid oxidation and ketone production in the liver [160]. Typically, FGF21 is not expressed in skeletal muscle, but its expression is induced by various stresses, including lipodystrophy, chronic muscular hyperinsulinemia, and mitochondrial dysfunction [161, 162]. FGF21 is an important metabolic regulator that maintains energy balance throughout the whole body [157].

### Skeletal muscle

In the skeletal muscle-specific AKT1 transgenic mouse model, FGF21 was found to be upregulated in gastrocnemius muscle and serum, suggesting that the expression of FGF21 is regulated by the PI3K/AKT1 signaling pathway, which is also related to the myofiber hypertrophy and insulin signaling [163]. In muscle-specific FGF21-KO mice, it was found that FGF21 is necessary for fasting-induced muscle atrophy and weakness. This protection effect can be attributed to the maintained protein synthesis rate of the muscle, which has also been validated in the FGF21 overexpression mouse model. Furthermore, this process of regulating muscle mass requires the involvement of the mitochondrial protein BCL-2 interacting protein 3 (BNIP3) which controls mitophagy flux. Inhibition of BNIP3 reduces the mitophagy flux, thereby protecting against FGF21-related muscle atrophy [164]. However, it remains controversial whether FGF21 is a key metabolic mediator of mitochondrial stress adaptation and ameliorates mitochondrial myopathy. In *Fgf21*-KO mice, the role of endogenous FGF21 in improving obesity resistance, blood glucose control, and hepatic lipid homeostasis related to muscle mitochondrial stress can be negligible [165]. This may be related to the loss of the FGF21-receptor cofactor, KLB, in the skeletal muscle. From another perspective, it is known that mitochondrial fusion protein optic atrophy 1 (OPA1) deficiency in skeletal muscle can induce mitochondria dysfunction and lead to muscle atrophy, and muscle-secreted FGF21 is upregulated in OPA1-deficient mice [166]. Importantly, FGF21 resists weight gain and insulin resistance induced by age and diet in young OPA1-deficient mouse models [166]. However, in OPA1-deficient mice, FGF21 inhibition restores almost all aging-related effects, such as systemic inflammatory response and premature death, indicating potential treatments targeting FGF21 in aging-related diseases [167].

### Adipose tissue

The secretion of FGF21 in muscle tissues also acts distally to modulate adipose tissue metabolism. Ectopic expression of UCP1 in skeletal muscles can activate the integrated stress response (ISR), improve substrate metabolism, and prolong lifespan. At the same time, a 5-fold increase in circulating FGF21 was observed in UCP1 transgene (UCP1-TG) mice. The results of treating C2C12 myoblasts with respiratory electron transport chain inhibitors, antimycin A and myxothiazol, and the uncoupler carbonyl cyanide 4-(trifluoromethoxy)phenylhydrazone (FCCP) suggest that the activation of ISR is closely associated with the expression of FGF21 [168]. Simultaneously, an increased browning of WAT was observed in UCP1-TG mice. In this case, the serum of transgenic mice to treat primary white adipocytes could upregulate the expression of UCP1. These results indicate that skeletal muscle can secrete FGF21, which acts on adipocytes to promote browning and regulate thermogenesis [168]. In addition, Irisin, an exercise-induced myokine was found to enhance heat generation in cooperation with FGF21, indicating that such interaction between myokines represents a thermogenic mechanism [159]. Adiponectin, an insulin-sensitizing hormone, is also thought to act downstream of FGF21 in the regulation of energy expenditure and insulin action [169]. In addition to thermogenesis and browning, FGF21 stimulates fat decomposition in mouse WAT during feeding [170].

As a cytokine secreted by multiple organs, the functions and mechanisms of FGF21 as a myokine are associated with multiple metabolic processes. It is an essential factor for muscle function and metabolism. Simultaneously, it promotes the browning of WAT and thermogenesis to protect the body from stimuli. The regulatory roles of FGF21 provide explanations for various diseases.

## Apelin

Apelin is both a myokine and an adipokine that is also expressed in different tissues, such as the heart, lungs, and kidney tissues [171, 172]. It is an endogenous ligand for the G protein-coupled receptor, APJ (apelin receptor, APLNR, or AGTRL1) [173]. Apelin was first defined as a myokine in endurance training research for obese individuals [174]. By establishing an *in vitro* primary culture of myoblasts from obese non-diabetic male subjects who had experienced an 8-week endurance training, researchers found that the level of apelin mRNA had a 2-fold increase in skeletal muscle and might act through autocrine and paracrine manners.

### Skeletal muscle

Apelin decreases during aging, suggesting a close relationship between apelin and age-related disorders such as muscle function damage. Specifically, during the aging process, this peptide may significantly improve muscle functions by inducing mitochondriogenesis, autophagy, and anti-inflammatory pathways in the muscle, as well as promoting regeneration ability by targeting muscle stem cells [175]. Mice deficient in apelin have significantly reduced insulin sensitivity and adiponectin levels, indicating that apelin is important in regulating metabolic homeostasis [176].

From a pharmacological perspective, previous studies have demonstrated that both acute and chronic apelin treatment ameliorate insulin resistance and improve muscle functions [176, 177]. In insulin-resistant mice, apelin improves mitochondrial biogenesis and enhances mitochondrial function through the AMPK pathway, indicating the potential of apelin in treating insulin resistance [178].

Specifically, apelin administration reduces fat mass, blood glucose, and plasma triglycerides in HFD mice, and the treatment improves mitochondrial biogenesis and oxidative capacity in the soleus, suggesting the role of apelin on muscle [178]. Besides, in C2C12 myoblast cells, apelin treatment promotes AKT phosphorylation and, hence, glucose uptake. In addition, a subgroup of chronic kidney disease (CKD) is associated with skeletal muscle atrophy. Administration of apelin in CKD mice significantly ameliorates weight loss and muscle atrophy, indicating its therapeutic potential [179].

### Other organs

Secreted apelin can also act on various organs besides skeletal muscles. For example, apelin secreted by the skeletal muscle can bind to APJ in vascular endothelial cells to promote their expansion. TEA domain transcription factor 1 (Tead1) is a novel regulator for the apelin promoter, and its overexpression in the muscle inhibits the secretion of apelin *in vivo*. The Tead1-apelin axis associates myofibers with endothelial cells and such a function partially explains the mechanisms of endothelial cell remodeling during muscle repair [180]. By using electrocardiogram (ECG) and other cardiovascular-related measurement methods in mouse hearts, endogenous apelin was found to reduce left ventricular load and increase cardiac contractility, suggesting its potential role in treating heart failure [181].

Together, apelin has a positive effect on muscle quality, function, and metabolism. It is associated with aging and can improve insulin resistance. It also helps regulate the interaction between muscles and the circulatory system.

## BDNF

Brain-derived neurotrophic factor is a member of the neurotrophins, a small protein produced by neurons [182]. It affects the survival and differentiation of central neurons and influences the neuromuscular system [183]. Besides, BDNF is also detected in peripheral tissues, including the liver, skeletal muscle, and adipose tissue [184]. Interestingly, gender differences are particularly noteworthy in BDNF research [185].

### Skeletal muscle

Normally, skeletal muscle can switch fuel sources between lipid and glucose oxidation during metabolic stress, and dysregulation of the flexibility might cause metabolic disorders. As a myokine induced by fasting, BDNF controls this metabolic reprogramming process through the AMPK/cyclic AMP (cAMP) response element-binding protein (CREB)/PGC-1α pathway in female mice, switching the main source to fatty acids during fasting. However, the expression of BDNF in the skeletal muscle does not rise in the same way in fasted male mice. Additionally, both BDNF expression and secretion are increased in glucose-depleted C2C12 myotubes, a muscle cell line isolated from a female mouse. These results indicate that BDNF is expressed in a sex-specific manner [185].

In 2009, BDNF was found to improve fatty acid oxidation in skeletal muscle through the AMPK pathway [186]. BDNF stimulates mitochondrial fission and clearance in skeletal muscle, indicating its importance in maintaining mitochondrial quality and function. In muscle-specific *bndf*-KO (MBKO) mice, increased mitochondrial number is probably due to the blocked clearance mechanisms. In addition, BDNF-deficient cells exhibit diminished respiratory reserve, suggesting that the mitochondria-related stress-buffering system has been compromised in these cells [187]. Correspondingly, impaired muscle autophagy was found in MBKO mice. Given that excessive autophagy can lead to muscle atrophy and worsen muscle disorders, further research is advisable to continue investigating the effects of BDNF on muscle autophagy [185].

BDNF is also necessary for the specification of muscle fiber type. BDNF muscle-specific KO mice show a shift in myofiber ratio from type IIB to IIX and a concomitant elevation of slow muscle-type gene expression. Conversely, overexpression of BDNF enhances the fast muscle-type gene program and increases the number of glycolytic myofibers [183]. These findings reveal its role in metabolic regulation through modulating muscle fiber type switch.

### The liver

Currently, our understanding of BDNF in the liver remains limited. In patients with alcoholism and hepatitis B-induced cirrhosis, BDNF levels are altered [188, 189]. By reducing the membrane TRKB-T1 (a truncated isoform of tropomysin related kinase B (TrkB)) protein, BDNF can ameliorate hepatic steatosis and diet-induced nonalcoholic steatohepatitis [190]. Under endoplasmic reticulum (ER) stress, BDNF has been found to suppress growth arrest- and DNA damage inducible gene 153 (GADD153) and SREBP-1c to protect hepatic cells from apoptosis and steatosis [191]. However, another study showed that in the whole population and alcoholics, BDNF levels are poorly associated with liver dysfunction, which needs further investigation [188].

In summary, as a member of the neurotrophic factor family, there have been extensive researches on the relationship between BDNF and the nervous system. BDNF also acts as an upregulated myokine after exercise, participating in the regulation of energy metabolism, especially fuel selection.

## Metrnl

Meteorin-like, a protein that is highly homologous with a neurotrophic factor called Meteorin, is also referred to as Meteorin-β, Cometin, or Subfatin [192]. It was first identified as a myokine as well as an adipokine by Rao *et al*., who revealed that Metrnl plays a vital role in metabolic regulation [193].

Compared to healthy individuals, serum Metrnl levels are lower in patients with obesity and T2D [194–197]. Additionally, multivariate logistic regression analysis revealed that high circulating Metrnl levels are significantly associated with declined risk of T2D, and low serum Metrnl levels are correlated with high blood glucose levels, high insulin resistance, and worsened glucose tolerance [198]. However, inconsistent results have also been reported. One meta-analysis concluded that circulating Metrnl does not change significantly in T2D patients [199]. Moreover, a study found that plasma Metrnl levels are higher in T2D patients [200]. It was proposed that this result might be due to the compensatory effect of restoring glucose tolerance [192]. However, in patients newly diagnosed with T2D, serum Metrnl levels are also significantly lower than normal [201].

As revealed by Rao *et al*. in PGC-1α4 transgenic mice, the expression of Metrnl can be induced in muscle by exercise [193]. PGC-1α4 is a splice isoform of PGC-1α, which is an upstream effector protein of Metrnl. Later, it was revealed that AMPK activation upregulates PGC-1α after exercise, increasing mitochondrial ATP production [202]. A study of nine male subjects found that high-intensity interval exercise increases the mRNA level of Metrnl in skeletal muscle [203]. In addition to exercise, bariatric surgery for T2D patients also affects Metrnl levels. Laparoscopic sleeve gastrectomy increases Metrnl levels, together with improved glucose and lipid homeostasis in patients [204, 205].

Exogenous Metrnl improves mitochondrial dysfunction induced by palmitic acid via the Sirt3-AMPK signaling axis [206] as well as being closely related to metabolism by affecting processes including insulin sensitivity, facilitating adipose tissue browning, and increasing energy expenditure in different organs [207]. An analysis of 182 subjects also found that the serum Metrnl levels are negatively correlated to the levels of total cholesterol, triglyceride, and low-density lipoprotein (LDL), indicating its effects on lipid metabolism [196].

### Adipose tissue

As for some other myokines such as irisin, Metrnl stimulates the white-to-brown shift of adipose tissues. However, Metrnl does not directly act on adipocytes. Instead, it depends on the eosinophil-mediated IL-4/IL-13 signaling cascade in alternately activated M2 macrophages. The activation of macrophages is essential for promoting the expression of anti-inflammatory and thermogenic genes in adipose tissues. Notably, the browning effect induced by Metrnl disappears in only one week [193]. In BAT, Metrnl might enhance thermogenesis by activating the UCP family. Fatty acid β-oxidation gene programs are also induced by Metrnl stimulation, such as carnitine palmitoyltransferase 1 (*Cpt1*) and acyl-CoA oxidase 1 (*Acox1*) [193]. However, another study showed that Metrnl is not associated with the white-to-brown shift of adipocytes or the expression of UCP1 in human samples [208].

Metrnl also regulates the differentiation of human adipocytes. It inhibits the differentiation of adipocytes as indicated by inhibited lipogenesis and decreased expression of PPARγ and markers of adipogenesis [208]. However, another study showed its ability to promote adipogenesis in mice via the PPARγ pathway [209]. This was validated in mesenteric adipose tissue [210]. These conflicting results demonstrate the need for additional research.

### Skeletal muscle

Electrical pulse stimulation and exercise trigger muscle contractions, which increase Metrnl levels *in vitro* and *in vivo*, respectively. Additionally, by inducing the calcium-dependent AMPKα2 pathway, Metrnl increases the phosphorylation of histone deacetylase 5 (HDAC5), thereby activating the transcription of glucose transporter type 4 (GLUT4) and thus promoting glucose uptake in the skeletal muscle [211].

Metrnl also induces AMPK- or PPARδ-mediated signaling, which improves insulin resistance and decreases inflammatory responses in skeletal muscle. Moreover, this signaling pathway induces fatty acid oxidation by upregulating related genes including fatty acid binding protein 3 (FABP3), acyl-CoA oxidase (ACO), and CPT1 [212].

### Other organs

Regarding the pancreas, Metrnl treatment ameliorates the reduced cell viability and insulin secretion caused by high glucose. It also activates the Wnt/β-catenin pathway in pancreatic islets, which inhibits apoptosis and promotes the proliferation of β-cells [213].

Metrnl also increases bone mass by reducing the activity of osteoclasts [214]. However, another work demonstrated that Metrnl does not affect healing tissue in terms of the amount of bone deposited and the structural parameters *in vivo*, whilst it does promote osteoblast differentiation *in vitro* [215].

Together, Metrnl expression is increased by exercise, which promotes the white-to-brown shift of adipose tissues via macrophages, indicating the complex mechanisms involved in increasing energy expenditure. Metrnl also improves glucose metabolism in skeletal muscle and preserves β-cells. However, its effects on adipocytes and bone remain contradictory, requiring further studies.

## SPARC

Secreted protein acidic and rich in cysteine, also named basement-membrane protein BM-40 or Osteonectin, was previously discovered in bone, adipose tissue, and many other tissue types [216–218]. It is a matricellular glycoprotein that mediates interactions between cells and proteins from the extracellular matrix (ECM) [219]. Adopting DNA microarray and bioinformatics tools, Aoi *et al*. first identified SPARC as a myokine in mice. Later, this was also validated in humans [220, 221].

Circulating levels of SPARC are increased in obesity and diabetes in both mice and humans, and mice with SPARC deficiency exhibit impaired glucose homeostasis and insulin secretion [222, 223]. However, one study also found that plasma SPARC levels are lower in obese women compared to the controls [224]. Exercise is proven to decrease serum SPARC levels in humans [225]. However, studies have also reported that exercise and muscle stretching stimulate SPARC expression and release from skeletal muscle [220]. A study on zebrafish also showed increased production of SPARC in muscle satellite cells (MuSCs, also named muscle stem cells) in response to exercise [226]. As in the case of the quantification of irisin levels, measurement of plasma SPARC levels might require more precise antibodies and refining of methods. Since SPARC is widely expressed in various tissues, the circulating level might also be influenced by different secretion sources, as regulated by different mechanisms. When studying the effects of exercise, exercise-induced changes in plasma volume should be considered, since these might negate the changes of the SPARC levels [224].

### Skeletal muscle

In mice, SPARC interacts with voltage-dependent calcium channels, which increases the phosphorylation of AMPK, promoting the expression levels of PGC-1α and thus resulting in elevated expression of GLUT4 [227, 228]. In this way, glucose uptake of skeletal muscle and glucose tolerance are improved by SPARC treatment which has also been shown to prevent the development of insulin resistance in mice [227]. Additionally, SPARC treatment increases the transition of glucose into glycogen in human skeletal cells, indicating its ability to improve glucose homeostasis, since skeletal muscle plays a major role in whole-body glucose uptake [227].

PGC-1α is a main mediator of mitochondrial biogenesis, mediating cellular responses to metabolic stresses such as exercise [229]. As previously mentioned, it is regulated by SPARC via AMPK phosphorylation and de-phosphorylation. Changes in ECM may also affect mitochondrial functions in muscle cells. SPARC has been shown to modulate ECM by the integrin-linked kinase/phosphorylated-GSK-3β pathway, which might indirectly regulate protein expression in muscle mitochondria [229].

During the development of obesity and T2D, adipose tissue appears between skeletal muscle fibers. Such adipose tissue is, therefore, defined as intramuscular adipose tissue (IMAT). IMAT is associated with insulin resistance and is probably involved in its development [230]. In aged mice, FGF-2 induces the expression of Fos-related antigen 1 (Fosl1), which, in turn, triggers the expression of miRNA-29a. This downregulates adipogenic inhibitor SPARC, causing fibro-adipogenic progenitors (FAPs) to convert to adipocytes and resulting in fat accumulation in skeletal muscle [231]. The FGF-2/miR-29a/SPARC pathway has also been validated in humans [231, 232]. The same group also revealed that insulin-like growth factor I (IGF-I) directly upregulates SPARC by activating its promoter, probably via a PI3K-dependent signaling pathway. Growth hormone can stimulate IGF-I systemically, which restores SPARC levels in skeletal muscle. Although growth hormone treatment fails to prevent IMAT formation, such a finding indicates potential treatment targets for insulin resistance [233].

### Other organs

In mouse adipose tissues, SPARC has been shown to inhibit the mitotic clonal expansion and differentiation of preadipocytes during adipogenesis by enhancing the Wnt/β-catenin signaling pathway [234, 235]. SPARC is markedly upregulated in adipose tissues in both obese rodent models and humans with obesity and also has been reported to improve insulin resistance in mice [217, 222]. SPARC promotes adipose tissue inflammation, and downregulation of SPARC by caloric restriction acts to reduce inflammation via the inhibition of its activation of the NACHT, LRR, and PYD domains-containing protein 3 (NLRP3) inflammasome [236, 237].

In the liver, SPARC deficiency triggers the deposit of free fatty acids, thereby increasing lipid droplets within hepatocytes [238]. Notably, nuclear translocation of SREBP-1c, an important transcription factor critical for the synthesis of fatty acids, has been observed in the absence of SPARC. As such, SPARC inactivation is associated with more severe liver steatosis and accelerated development of NAFLD-related hepatocellular carcinomas (HCCs) [238].

In summary, SPARC is upregulated by exercise, which promotes glucose metabolism in the skeletal muscle and inhibits IMAT. It is also associated with adipogenesis, hepatic lipid metabolism, and vascular systems. Further study might also explore the effects of SPARC on ECM in adipose tissue, which affects cellular activities indirectly.

## BAIBA

As a type of metabolite from skeletal muscle, BAIBA is an amino acid metabolite [239]. Although myokines are defined as skeletal muscle-secreted proteins or peptides, recent studies have included small metabolites in myokines [22, 30].

In 1951, BAIBA was first discovered in human urine [240]. BAIBA has two enantiomers in the human body, D-BAIBA (R-BAIBA) and L-BAIBA (S-BAIBA), which have potentially different biological functions [241, 242]. A study found that D-BAIBA is predominant at baseline, and the serum levels of both D- and L-BAIBA elevate after exercise [243]. However, other researches reported that L-BAIBA is the major enantiomer in plasma [241, 244]. D-BAIBA and L-BAIBA are produced from thymine and valine, respectively [241]. Besides, they are also expressed by different organs, and L-BAIBA, instead of D-BAIBA, is generated by muscle mitochondria [241]. As an amino acid metabolite, BAIBA is considered a muscle-signaling metabolite regulating multiple metabolic processes.

### Adipose tissue

The most widely studied role of BAIBA is its regulation of the browning effect of WAT. In 2014, BAIBA was found to act as a novel mediator for WAT browning and thermogenesis under the intervention of exercise and PGC-1α treatment. In this study, BAIBA was seen to promote the expression of BAT-specific genes in WAT through a PPARα-dependent pathway, demonstrated experimentally both *in vivo* and *in vitro* [22]. Proteins, including UCP1, mitochondrial biogenesis transcription coactivator PGC-1α, and cell death-inducing DNA fragmentation factor-α (DFFA)-like effector A (CIDEA), are upregulated, mediating mitochondrial energy consumption and increasing the thermogenesis of adipose tissue [22]. These changes might lead to inhibited body fat accumulation triggered by BAIBA [242]. However, UCP3, the main subtype of the UCP family expressed in skeletal muscle, has no clear connection with the expression of BAIBA [245].

### The liver

BAIBA is found to promote hepatic fatty acid β-oxidation and is negatively correlated with triglyceride and cholesterol levels in the circulation [22]. It might be achieved by the activation of enzymes responsible for oxidating free fatty acids and the assembly of very low-density lipoproteins (VLDLs) in the liver [242]. VLDLs are synthesized by hepatocytes and transport molecules, including triglycerides and cholesterol, from the liver into peripheral tissues [246]. One of the major features of diabetic dyslipidemia is increased VLDL production [247]. It is claimed that BAIBA regulates lipid metabolism by influencing the production of liver VLDL [242]. BAIBA could increase fatty acid oxidation in the liver and thus prevent fat accumulation in a leptin-dependent manner [248]. In addition, by treating HepG2 cells with BAIBA, it was found that BAIBA can reduce triglyceride synthesis in the liver by activating the AMPK pathway. This provides evidence that BAIBA is likely to regulate lipid accumulation caused by liver ER stress [249]. Besides lipid metabolism, BAIBA also reduces ER stress in hepatocytes with insulin resistance and protects the cells from apoptosis [249].

### The bone

Over recent years, understanding of the regulation of bone tissue by BAIBA has increased. Osteocyte death is regulated in multiple ways, including the actions of ROS to lead bone cells to death by the breakdown of mitochondria. In a mouse model of osteocyte apoptosis, adding L-BAIBA to drinking water significantly protects bone mass through the Mas-related G protein-coupled receptor type D (MRGPRD) [250]. Correspondingly, as age increases, the expression of MRGPRD decreases along with declined BAIBA protection ability, which partly explains osteocyte damage during aging. In addition, BAIBA decreases bone loss with unloading in mice, and L-BAIBA treatment can work together with sub-optimal mechanical loading to promote bone formation [251].

In summary, although BAIBA is a type of amino acid metabolite, it also can be classified as a myokine, regulating metabolic processes. BAIBA promotes the browning of WAT, thermogenesis, and free fatty acid oxidation in adipose tissue. At the same time, it also has a positive effect on lipid metabolism in the liver. It has also been demonstrated that BAIBA can protect the survival of bone cells and promote bone formation.

## Musclin

Musclin, a peptide belonging to natriuretic peptides (NPs), is a myokine involved in the regulation of a variety of metabolic processes [252, 253]. It was originally discovered using a viral-based signal-trap strategy in 2003 and was initially named osteocrin (OSTN) [254]. It regulates osteoblast functions, osteocytogenesis, and bone development [255, 256]. One year later, this peptide was confirmed as a myokine and renamed Musclin, and scientists revealed its function in reducing insulin-triggered glucose uptake and glycogen synthesis in the skeletal muscle [257]. Including the N-terminal 30-amino acid signal peptide, Musclin cDNA consists of a total of 130 amino acids. At the same time, the Musclin sequence contains a sequence homologous to the NP family and a serine protease cleavage site, KKKR [257]. It has been demonstrated that production of Musclin in the muscles is driven by Ca^2+^-dependent AKT1 activation and the release of *Musclin* transcription from inhibition from FoxO1 [258].

The NP family is mainly divided into three subtypes, namely atrial NP (ANP), B-type NP (BNP), and C-type NP (CNP). These NPs play regulatory roles in blood pressure and ventricular hypertrophy and participate in metabolism [252]. Currently, there are three known NP family receptors: NP receptor-A (NPRA), NPRB, and NPRC. In previous research, it was found that Musclin can competitively bind to the clearance receptor, NPRC, thereby affecting the level of ANP [259]. Given that NPs can activate the cyclic guanosine monophosphate (cGMP) signal, which rescues mitochondrial dysfunction, the indirect regulation of ANP and cGMP by Musclin is likely to mediate its effect on mitochondrial function [260, 261]. Additionally, there is also a correlation between Musclin and CNP. Increased plasma CNP level was observed in mice overexpressing Musclin, improving cardiac dysfunction and myocardial fibrosis [262, 263]. In bone-related research, it has been found that periosteal osteoblasts can produce Musclin to increase the levels of CNP and promote the proliferation and maturation of chondrocytes, thereby promoting bone formation [264].

As an important myokine, the protective and positive effects of Musclin on muscle tissue have been partially studied. The level of systemic Musclin is significantly upregulated after exercise, thereby indirectly regulating the concentration of ANP to enhance exercise endurance by improving mitochondrial biogenesis [258]. From a pathological perspective, Musclin plays a role in ameliorating muscle atrophy and muscle fibrosis induced by cancer cachexia. As such, Musclin may have the potential to treat muscle atrophy and improve resistance to injury [263, 265]. Also, the *Musclin* (*Ostn*) gene is highly expressed in the Tafazzin knockdown (TazKD) mouse, an experimental model exhibiting dilated cardiomyopathy, which is considered to provide a compensatory response to myocardial cell damage [266]. Moreover, sarcolemal ATP-sensitive potassium (K_ATP_) channels were noted to control the energy expenditure of skeletal muscle by controlling the excitability of cell membranes and related functions. In transgenic mice with skeletal muscle-specific disrupted K_ATP_ channels, Musclin secretion is elevated and is associated with increased mobilization of fatty acids. These data suggest that Musclin links the K_ATP_-dependent energy expenditure in skeletal muscle with the mobilization of fat [267]. Interestingly, a recent study found that Musclin inhibits the proliferation of FAPs and promotes apoptosis of the cells by upregulating filamin A interacting protein 1-like (FILIP1L). Exercise induces the production of Musclin, which reduces FAP frequency and adipose formation in the disused or injured muscle. This study revealed the regulatory role of Musclin in muscle atrophy or injury [268].

As a systemic metabolic disease, T2D is closely related to insulin resistance and other abnormal metabolic events, such as altered glucose and lipid metabolism. To date, the role and mechanism of Musclin in the development of obesity and T2D remain elusive. Musclin expression is elevated in T2D and it impairs glucose metabolism in myocytes [257]. Some studies suggest that the expression of Musclin is induced by exercise in both humans and mice [258, 268, 269]. However, a study claimed that Musclin levels are downregulated by exercise intervention [270], which was demonstrated by our group [253]. Musclin has also been shown to be positively correlated with insulinemia, insulin resistance, and high visceral fat in human study [271]. Hence, Musclin is considered a critical negative regulator of systemic energy homeostasis.

Recently, research from our laboratory uncovered an unexpected role of Musclin in the regulation of thermogenesis in beige adipose tissue and systemic energy balance under both physiological and pathological conditions [253]. Muscle expression and circulating levels of Musclin are elevated in mice housed at thermoneutral temperature and markedly decreased in mice upon cold exposure. These findings suggest that Musclin is a cold-sensitive myokine and may play a role in regulating thermogenesis and maintaining body temperature. In line with this hypothesis, muscle-specific overexpression of Musclin reduces the thermogenesis of adipose tissue, making mice more susceptible to HFD-induced obesity and metabolic disorders. In contrast, muscle-specific inactivation of Musclin promotes thermogenesis and improves systemic glucose homeostasis. Further studies revealed that inguinal WAT (iWAT) is the primary target of Musclin, which contains thermogenic beige adipocytes. Beige adipocytes are an inducible form of thermogenic adipocytes that arise within WAT in response to lower ambient temperature. Elevated circulating Musclin acts on iWAT to inhibit metabolic and thermogenic gene expression programs, leading to more severe HFD-induced obesity and metabolic dysfunction. Musclin blockade by either muscle-specific KO or Musclin-neutralizing antibody promotes beige fat thermogenesis and improves systemic glucose metabolism. This is achieved by upregulating the expression of genes involved in energy metabolism and thermogenesis, including fatty acid and glucose metabolism genes, UCP1, as well as subunits of mitochondrial oxidative phosphorylation (OXPHOS) complexes.

Using proximity-dependent biotin identification (BioID) assay technology, transferrin receptor 1 (Tfr1) was identified as a new receptor for Musclin on the plasma membrane of adipocytes to antagonize cAMP/protein kinase A (PKA)-dependent thermogenic induction [253]. Tfr1 was shown to play an important role in adipose tissue thermogenesis and is associated with insulin resistance and mitochondrial dysfunction [272]. The discovery of the new receptor provides critical mechanistic insights into the regulatory mechanism of Musclin in adipose tissue metabolism and possible new ideas for clinical application.

Overall, the positive or negative effects of Musclin on organisms are complex and remain to be fully elucidated. Musclin has been demonstrated to play a positive role in muscle function and bone formation. However, as a critical negative regulator of beige fat thermogenesis and energy expenditure, it acts synergically with other thermogenesis activators to link muscle bioenergetic and nutrient-sensing functions to the control of systemic metabolic homeostasis under both physiological and pathophysiological conditions through muscle-beige fat interactions.

## Dkk3

Dickkopf genes represent a small gene family consisting of four members (Dkk1–4) and a Dkk3-related gene, Dkkl1 (soggy) [273]. Acting as antagonists, Dkks participate in the regulation of the Wnt pathways, which mediate a wide range of cellular activities and are involved in T2D development [274, 275]. Dkks suppress Wnt signaling by inhibiting the coreceptors LDL receptor-related protein 5 (Lrp5) and Lrp6, as well as binding to transmembrane proteins Kremen1 and 2 [273]. They are essential to the developmental processes in vertebrates and are also notably involved in inflammation, atherosclerosis, cancer, and Alzheimer’s disease in human adults [276].

Among them, plasma Dkk1 shows increases in T2D patients. This is partly due to platelet activation. Improved control of blood glucose levels also results in reductions in elevated circulating levels of Dkk1 [275]. Dkk1 may be activated by the PPARγ signaling pathway, inhibiting the Wnt signal and promoting adipogenesis [277]. Dkk2 is also a Wnt antagonist. One study has shown that inhibition of Dkk2 improves glucose tolerance and reduces basal blood glucose levels. Contrastingly, Dkk2 loss triggers increased Wnt activity and GLP-1 secretion, indicating that Dkk2 plays an indirect role in glucose tolerance [278]. Dkk4 is closely related to cancers where its expression is regulated differently in distinct types of cancers. In this case, it is not only involved in the development of tumors but also osteoblastogenesis and schizophrenia [279]. Differing from other members, Dkkl1 is expressed in developing spermatocytes and in the trophectoderm/placental lineage, indicating its importance in fertilization and development [280–282].

Being a novel myokine, Dkk3 is distinct, as the only one enriched in the skeletal muscle [283, 284]. A transcript of the *Dkk3* gene was found to be expressed in the skeletal muscle in 2000 [285]. It has also been reported to serve as a potential noninvasive plasma biomarker for Alzheimer’s disease [286] and to play a role in renal diseases and CVDs [287]. Additionally, it participates in bone regeneration, where Dkk3 levels are decreased after exercise [288]. Importantly, Dkk3 also participates in the development of age-related muscle atrophy [284, 289]. Obesity and diabetes not only result in insulin resistance and dysregulated metabolism but also impair the maintenance and regeneration of the muscle. Therefore, it is necessary to broaden the scope to include the study of the related mechanisms of diabetic myopathy [290].

As a significant complication of T2D, diabetic myopathy also promotes the progression of other diabetic complications, which are often ignored when studying the development of T2D [290]. During the disease progression, muscle progenitor cells are negatively affected, which impairs the regeneration capacity of skeletal muscles, thus contributing to the deterioration of muscle health [291]. MuSCs play a vital role in muscle regeneration [292].

Emerging evidence has demonstrated that epigenetic changes are closely associated with the development of T2D [293], central to this being the factor of chromatin remodeling [294]. Such a process is mediated by chromatin remodeling complexes, among which the switching defective/sucrose nonfermenting (SWI/SNF) chromatin-remodeling complex is essential in various cellular activities, including energy metabolism and nutrient signaling [295]. The Brahma-related gene 1/Brahma homolog (BRG1/BRM)-associated factor 60 (Baf60) subunit acts as a linker between the core complex and tissue-specific transcription factors in the SWI/SNF complex [295]. It has three members, Baf60a, Baf60b, and Baf60c, which are distributed differently and have distinct regulatory mechanisms [295]. Among them, Baf60c is highly expressed in the skeletal muscle [18, 296].

Expression levels of Baf60c are significantly decreased in skeletal muscles from both diabetic patients and mice, indicating a role of Baf60c in the development of diabetic myopathy [296, 297]. Using Baf60c muscle-specific KO (BcMKO) and transgenic (MCK-Bc) mice, it was attempted to explore this possibility and the underlying mechanisms. In BcMKO mice, muscle regeneration after injury was indeed found to be impaired. Subsequent RNA sequencing, gene ontology (GO), and qPCR analysis revealed that loss of Baf60c triggers transcriptional reprogramming, which promotes the expression of inflammation-related genes and suppresses injury-induced elevation of genes related to muscle differentiation. However, MuSC-specific Baf60c KO does not produce any significant effect on muscle regeneration as BcMKO. Using shRNA targeting Baf60c, a stable muscle cell line with Baf60c knockdown was generated. Conditioned medium from Baf60c-deficient myotubes was harvested, which attenuated the differentiation of muscle stem cells. Secreted-protein encoding genes were analyzed, among which *Dkk3* was identified to be the one most significantly upregulated. These results indicated that mature myocytes with decreased Baf60c might affect MuSCs by upregulating the production and secretion of Dkk3. The ability of Dkk3 to attenuate muscle regeneration was subsequently verified both *in vitro* and *in vivo*. In contrast, *Dkk3* knockdown in adult BcMKO mice rescues the muscle regeneration defect, demonstrating the regulating function of Dkk3. Similarly, myofiber-specific Baf60c (MCK-Bc) transgenic expression inactivates Dkk3 and improves muscle regeneration in mice. At the mechanistic level, BcMKO leads to an attenuation of the AKT/mTOR signaling pathway in regenerating skeletal muscle. Furthermore, treatment of the Dkk3 protein fused with the crystallizable fragment (Fc) domain of immunoglobulin G suppresses the activities of the AKT/mTOR signaling pathway in regenerating myotubes. Through further analysis of chromatin activity utilizing the assay for transposase-accessible chromatin using sequencing (ATAC-Seq) and cleavage under targets and tagmentation (CUT&Tag)-Seq, in combination with chromatin immunoprecipitation (ChIP)-qPCR and co-immunoprecipitation (Co-IP) techniques, it was found that Baf60c physically interacts with transcription factor sine oculis homeobox 4 (Six4) to suppress *Dkk3* gene transcription in myocytes.

Together, in myofibers, Baf60c regulates Dkk3-mediated paracrine signaling by physically interacting with Six4, upregulating the activity of the AKT/mTOR signaling pathway, which controls the regenerative capacity of MuSCs. Moreover, in obese human individuals, the skeletal muscle expression and circulating levels of Dkk3 are increased. Knockdown of *Dkk3* in the skeletal muscle alleviated the impaired muscle regeneration and contraction in obese mice. These data suggest the critical role of Baf60c-Dkk3 axis in T2D-associated skeletal muscle diseases [296]. The work provides the molecular basis for developing novel strategies for the clinical prevention and treatment of obesity and T2D-associated decline in skeletal muscle regeneration capacity and muscle mass.

## Conclusions

**Figure 2:**
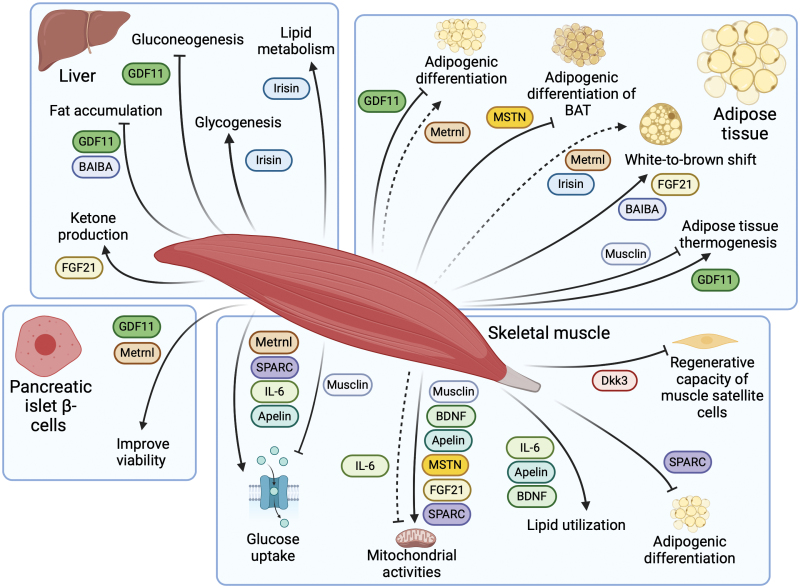
Metabolic effects of myokines on organs. Schematic diagram summarizing the main metabolic effects of IL-6, irisin, MSTN, GDF11, FGF21, apelin, BDNF, Metrnl, SPARC, BAIBA, Musclin, and Dkk3 on organs, including skeletal muscle, adipose tissue, liver, pancreatic islets. These myokines are secreted by skeletal muscle cells, which then act on organs, participating in the regulation of energy expenditure and metabolism. In terms of specific effects, the dotted line indicates speculation and/or controversy. BAT, brown adipose tissue; WAT, white adipose tissue. Created with BioRender.com.

**Figure 3:**
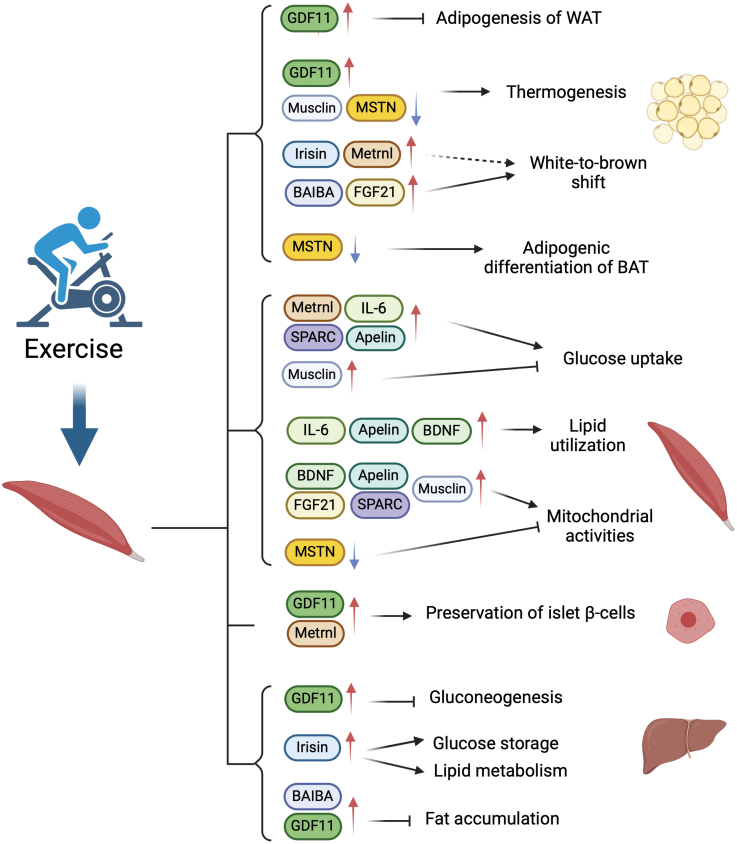
Physical exercise mediates the regulation of myokines. Schematic diagram of physical exercise-mediated regulation of myokine expression levels, summarizing the effects of exercise on major metabolic organs, including adipose tissues, skeletal muscle, β-cells, and liver. The levels of IL-6, apelin, irisin, GDF11, Metrnl, SPARC, FGF21, BDNF, and BAIBA increase after exercise, and the level of MSTN decreases after exercise. Notably, the level of Musclin has been reported to increase or decrease upon exercise depending on the experimental conditions. MSTN, myostatin; BAT, brown adipose tissue; WAT, white adipose tissue. Created with BioRender.com.

Besides the main role of skeletal muscle in exercise [17], many muscle-secreted factors have been identified and studied over recent years, confirming skeletal muscle to act as an important endocrine organ. Muscle-secreted factors, namely myokines, are synthesized and released by muscle cells, which exert effects on organs and tissues throughout the body, regulating metabolic homeostasis. Since the hypothetical “hypoglycemic factor” was proposed in 1961 [298], emerging research results have been achieved in this field, which greatly promote our understanding of muscle biology and exercise-induced human health [299] (Fig. 1). Lifestyle-related interventions, especially exercise interventions, have been proven to affect myokine levels, producing beneficial effects on preventing diseases, promoting health, and improving resilience [300]. Sources, functions, and molecular mechanisms of classic myokines, such as IL-6, have been established by abundant experimental evidence. Interactions between organs are now receiving increasing attention. For example, Kirk *et al*. summarized that myokines serve as bridges connecting muscle tissue with adipose tissue and bone tissue [301]. In this review, we summarize the effects of twelve representative myokines on different organs (Fig. 2), emphasizing their role in interorgan communication, which is important in systemic metabolic balance. We also conclude the regulatory role of exercise on these myokines (Fig. 3).

Up to now, very few myokines have been thoroughly studied, and most of their functions remain to be investigated. Due to the advancement of technologies, hundreds of novel myokines have been identified [302]. In 2006, the first human skeletal muscle secretome was produced by computational analysis [303]. A total of 153 skeletal muscle-produced secretory proteins were identified and a novel approach for conditioned secretome analysis was described by Yoon *et al.* [304]. Additionally, 635 skeletal muscle-produced secretory proteins during the differentiation of muscle cells were identified and quantitatively analyzed in 2010 [305]. However, how to effectively find the influential and decisive ones still requires further exploration.

Contributing to myokine regulation, specific mechanisms of some exercise interventions remain elusive. Acute and chronic exercise, aerobic and anaerobic exercise, and resistance exercise may induce different effects of myokines [79]. Hence, further studies are required to reveal the regulatory mechanisms. In terms of application, myokines also serve as possible auxiliary diagnostic indicators and potential therapeutic targets for diseases including obesity, T2D, CVDs, cancers, and neurodegenerative diseases. Further translational research is required, which is expected to result in the development of new treatments. Of course, the research related to myokines goes far beyond the above-described aspects, and there are also many unknown directions waiting for us to explore.

## Future directions

It is of note that, in addition to proteins and peptides, skeletal muscle also produces and secretes other factors, such as RNA and small metabolites, to regulate metabolism. Recently, a review named secreted non-coding RNAs “RNAkines”, and identified skeletal muscle-secreted non-coding RNAs like miR-22 and miR-133 as participators in the regulation of myogenesis and insulin sensitivity, indicating their role in metabolic regulation [306]. It has been reported that adipocytes secrete lipids to control interorgan communication, mediating metabolic homeostasis [307]. Recently, Hu *et al.* identified the first muscle-secreted lipid (named lipokine), 1,2-dilinoleoyl-*sn*-glycero-3-phosphocholine (DLPC), which can induce the browning of WAT via lipid peroxidation-mediated p38 activation in male mice, thus significantly improving HFD-triggered obesity [308]. Therefore, it is time to reconsider the definition of “myokines”, which was first defined in 2003 as proteins and peptides secreted by the skeletal muscle that exert their effects on other cells or organs of the body [21]. BAIBA, an amino acid generated by exercise muscle, was defined as a myokine that induces white fat browning and hepatic β-oxidation in 2014 [22]. In addition, lactate, a muscle-derived metabolite that has been shown to participate in the regulation of a variety of physiological functions, was also defined as myokine and exerkine [309]. Therefore, other muscle-secreted non-protein and non-peptide factors, including amino acids (such as glutamine [310]), lipids (such as DLPC [308]), and RNAs (such as miRNAs), may also be defined as myokines in future studies.

Focusing on previously studied pathways and molecules that are associated with metabolic processes, researchers then went on to identify several myokines. For example, PGC-1α is known for regulating exercise-induced effects on muscles, which led to the identification of myokines including irisin and BAIBA [22, 64]. Another way to start identifying new myokines is to study extracellular vesicles. These are important means for the transportation of secreted proteins, providing a narrower and more precise range of candidate proteins [302].

## Current challenges and possible solutions

Like other research fields, the quick advancements of myokine research in the past two decades heavily relied on the development and application of new techniques. High throughput techniques can be used for the enrichment and quantification of candidate proteins (Box 1). Global RNA sequencing of skeletal muscle was conducted to identify potential novel muscle secretion factors under the exercise intervention [221]. Multi-omics approaches (Box 2), including proteomics and metabolomics, enable the discovery of new myokines and the measurement of their expression levels [311]. Recently, the capacity and accuracy of omics analysis have been significantly increased, especially aided by the low input-protein mass spectrometry. The technique offers information on protein identity, structure, and dynamics [312], and enables an unbiased, hypothesis-free analysis of a large number of proteins [302]. By integrating nanoparticle protein coronas with liquid chromatography-mass spectrometry, the efficiency of proteomic profiling can be further improved [313]. In addition, combined use of metabolomics and lipidomics also provides a pathway for finding muscle-secreted metabolites [314]. By combining whole-body multi-tissue expression data, the hybrid mouse diversity panel (HMDP), a population-based method, might be of particular use in the discovery of novel myokines [315]. The development of microproteomics also provides us with a more in-depth analysis of information about muscle single cells and muscle cell subpopulations [316]. For example, Li *et al.* described a method called Expansion Proteomics (ProteomEx), which combines mass spectrometry-based proteomics with hydrogel-based tissue transformation, achieving proteome profiling with a lateral resolution of 160 µm [317]. Combining the results obtained from multiple analyses can greatly enhance the study effectiveness. For example, differential gene expression profiling is a technique used to identify potential molecule candidates. This technique is combined with the results of potency assay testing to increase the accuracy of the findings. This method has shown promising results, achieving an excellent *in vitro* hit rate of 18%, and an *in vivo* hit rate of 9% [318].

Box 1 **Challenges in the field**

- How to discover and identify more potential myokine candidates
- How to more directly determine the sources of cytokine secretion
- How to define the roles of myokines in organ-organ crosstalk
- How to better trace myokines in the metabolic cycle
- How to more efficiently identify cell type-specific receptors for myokines

Box 2 **Potential solutions**

- New multi-omics technology, such as microproeteomics technology and single-cell RNA sequencing (scRNA-Seq)
- Advanced technologies for the identification of protein–protein interaction, such as BioID and TurboID
- New platforms for antibody production and protein quantification
- Dynamic imaging and tracing systems for tissue-specific secreted proteins *in vivo*

Since most myokines affect biological processes systemically, the potential muscle-tissue crosstalk mediated by myokines is very important. However, the complexity of myokine-related research is constrained by many factors. Firstly, a myokine may be secreted by multiple secretory organs as we summarized in Table 1, so it is important to determine the source of cytokines. In addition to the sources from different tissues, skeletal muscle consists of multiple cell types, including myocytes, immune cells, and fibroblasts [339]. It is thus difficult to define the source of cytokines even within skeletal muscle (Box 1). Clarifying such a source is not only important for understanding the related mechanisms but also for the development of therapeutic aspects of the treatment of metabolic diseases. Sequencing in different tissues or organs helps us better understand the distribution of myokines. New techniques such as cell type-selective secretome profiling *in vivo* help match cell types and secreted proteins and polypeptides, and thus contribute to the precise identification of cellular sources for myokines [340] (Box 2). Secondly, a myokine has the potential to impact various organs and tissues, which is illustrated in Fig. 2. Hence, labeling techniques are important for tracing myokines, which thereby helps reveal their biological roles. Traditional protein tracing techniques are achieved through isotope labeling of small metabolites such as lactic acid [341]. However, their specificity does not satisfy the needs of myokine research. Observation of real-time changes, even the dynamic process of myokine secretion is needed to trace myokines. In 2020, Sung *et al*. achieved fluorescence tracking and capture of exosomes in living cells in 2-dimension (2D) and 3-dimension (3D), enabling more intuitive observation of exosomes as a small but significant class of myokine [342]. By selectively labeling the proteins transported through classical secretion pathways through the catalytic action of protein transport protein Sec61 subunit β (Sec61b)-TurboID, dynamic tracking and identification of tissue-specific secreted proteins in the blood circulation of living mice were achieved [343]. This provides a good means for tracking myokine in blood circulation.

**Table 1:** Sources of myokines from metabolic organs. Other than skeletal muscle, many myokines are also produced by other metabolic organs.

Myokine	Tissue
IL-6	Skeletal muscle [39]
	Adipose tissue [319]
	Neuronal cells [320]
Irisin	Skeletal muscle [64]
	Adipose tissue [321]
	Liver [321]
	Neuronal cells [322]
MSTN	Skeletal muscle [1]
	Adipose tissue [114, 323]
GDF11	Skeletal muscle [324]
	Liver [325]
FGF21	Skeletal muscle [163]
	Adipose tissue [319]
	Liver [326]
Apelin	Skeletal muscle [174]
	Adipose tissue [327]
	Liver [328]
	Neuronal cells [329]
BAIBA	Skeletal muscle [22]
BDNF	Skeletal muscle [186]
	Adipose tissue [330]
	Liver [331]
	Neuronal cells [332]
Metrnl	Skeletal muscle [193]
	Adipose tissue [333]
	Liver [334]
	Neuronal cells [207]
SPARC	Skeletal muscle [220]
	Adipose tissue [326]
	Liver [335]
	Neuronal cells [336]
Dkk3	Skeletal muscle [284]
	Adipose tissue [337]
	Liver [337]
Musclin	Skeletal muscle [257]
	Neuronal cells [338]

Skeletal muscle, adipose tissue, liver, and neuronal cells are the major metabolic organs.

Antibody techniques are important for the quantification of myokines (Box 1), which is the key factor contributing to the controversial conclusions in previous studies on myokines such as irisin, SPARC, and GDF11. Good antibody technology is necessary for neutralizing target proteins, detecting circulating protein levels, or imaging techniques to help better identify targeted myokines *in vivo*. Additionally, the development of antibodies also benefits the application of myokines as potential therapeutic targets (Box 2). The fusion of myokines with antibodies or antibody fragments may also allow targeted delivery, thereby enabling the improvement and efficacy of pharmacokinetics [344]. Receptor identification is also critical for elucidating the downstream mechanisms of myokines. The discovery of receptors for secreted proteins has been challenging in the field (Box 1). Remarkably, the biotin-based proximity labeling BioID technique enables the detection of weak and/or transient protein–protein interactions in a more sensitive way [345] (Box 2). Using this BioID assay in combination with advanced mass spectrometry for protein identification, a recent study developed a feasible approach to solve this long-standing challenge in the field [253]. Tfr1 was successfully identified as the new receptor for Musclin in beige adipocytes. This discovery then paved the way to elucidate the signaling pathway mediating the regulation of thermogenic metabolism and systemic energy homeostasis by Musclin.

In summary, myokines play important roles in the regulation of energy metabolism and the pathogenesis of obesity and T2D. Although our understanding of myokine biology is still incomplete, emerging research advancements have already provided insightful prospects to explain metabolic processes in the human body and new biomarkers for the development of therapeutic drugs. We anticipate gaining further valuable insights into myokine-related research through the development and application of more innovative techniques in the near future.
